# Epidermal inclusion cyst of the thyroid: a rare case of a nodule-like structure at ultrasound

**DOI:** 10.1259/bjrcr.20200038

**Published:** 2020-06-09

**Authors:** Peter Lauffer, Joost van Schuppen, Christiaan F. Mooij

**Affiliations:** 1 Department of Pediatric Endocrinology, Emma Children’s Hospital, Amsterdam UMC, University of Amsterdam, Amsterdam, the Netherlands; 2 Department of Radiology and Nuclear Medicine, Amsterdam UMC, University of Amsterdam, Amsterdam, the Netherlands

## Abstract

An epidermal/(epi)dermoid cyst of the thyroid is a rare cause of an intrathyroidal mass. At radiological evaluation, it may initially be misinterpreted as a thyroid adenoma or carcinoma. We present a case report of a 15-year-old boy, who was evaluated because of a neck mass which caused globus pharyngeus and pain at swallowing. Ultrasound examination revealed a hypoechoic nodule-like structure in the left thyroid lobe. Aspiration of the nodule yielded white fluid. Cytological evaluation confirmed the diagnosis of an epidermal inclusion cyst of the thyroid.

## Clinical presentation

A 15-year-old boy with no medical history was referred to the paediatric endocrinologist because of a neck mass since 3 weeks. At ultrasound evaluation in a general hospital, a thyroid nodule of 2.0 cm wide, 2.8 cm deep and 3.2 cm tall was reported. He had globus pharyngeus and pain at swallowing; however, the complaints resolved spontaneously after 1 week. At physical evaluation, there was a smooth, painless lump palpable in the left thyroid lobe. Biochemical evaluation revealed euthyroidism (thyroid stimulating hormone 2.4 mU/L (local reference: 0.5–5.0 mU/L) and FT4 13.7 pmol/L (local reference: 12.0–22.0 pmol/L)). Calcitonin and thyroglobulin levels were normal as well.

## Imaging findings

**Figure 1.**
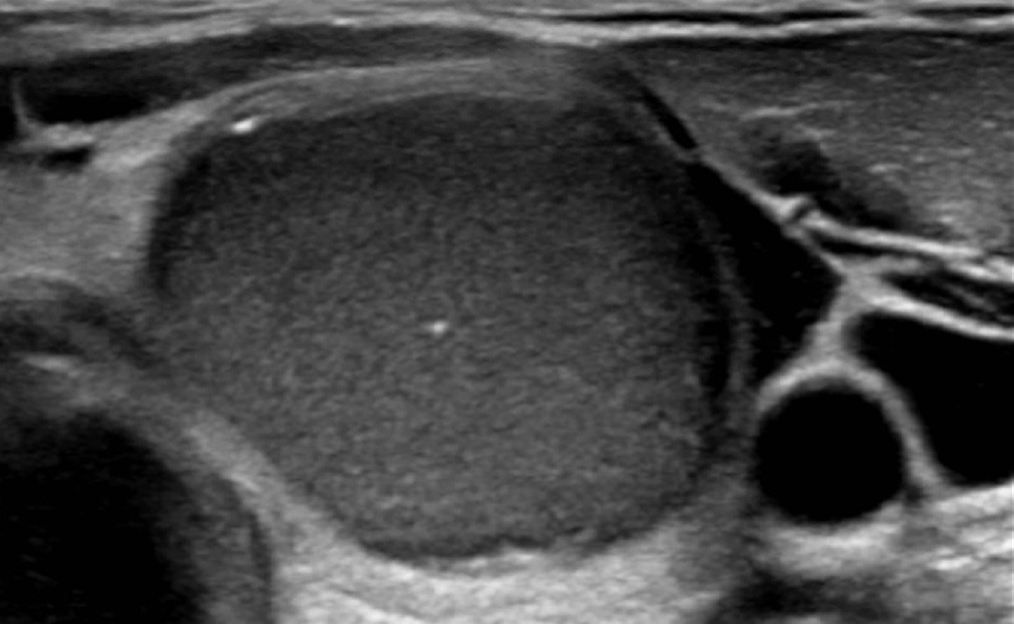
Transverse sonographic image of the thyroid gland. In the left thyroid lobe, there is a hypoechoic, well-demarked lesion. Macrocalcifications are ventral and in the middle of the cyst.

An additional thyroid ultrasound (Figure 1) showed a solid and homogeneous hypoechoic lesion with a well-defined uninterrupted border. Its shape was taller than wide (2.0 cm wide, 2.8 cm deep and 3.4 cm tall). Two macrocalcifications were seen in the lesion. Doppler signal was absent. Upon compression, there was elasticity to some degree, but without fluctuation. In the left cervical region, there was a lymph node of normal size, however without a normal fatty hilum, and with internal calcifications and a heterogeneous echotexture. Based on ultrasound evaluation of the thyroid and lymph node, the differential diagnosis included a thyroid adenoma, thyroid carcinoma and thyroidal epidermal cyst.

## Investigations

**Figure 2.**
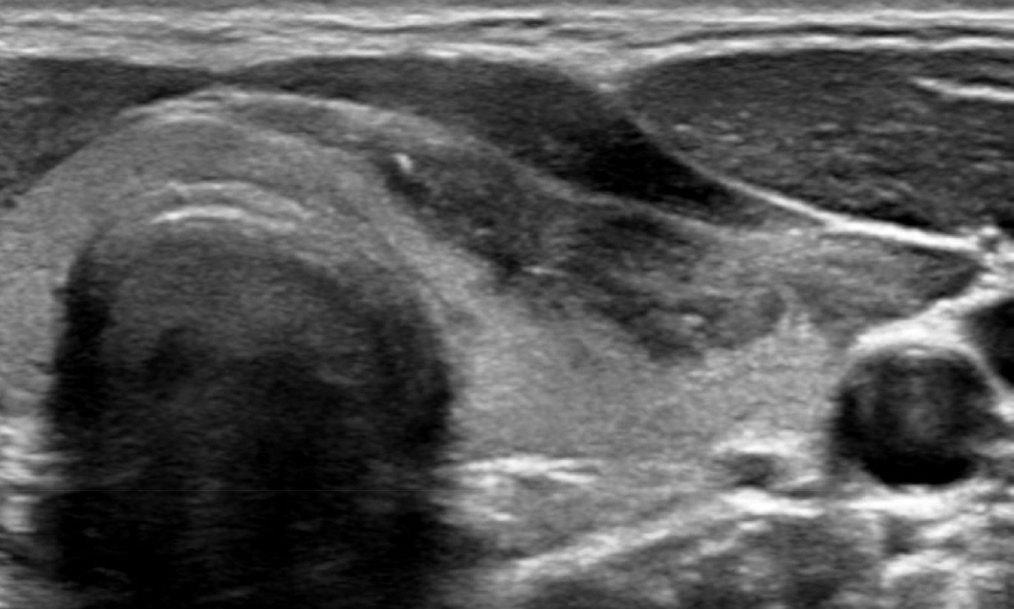
After fine-needle aspiration biopsy, only the cystic wall is left, which contains calcifications.

Based on the findings of the thyroid ultrasound, a thyroid carcinoma could not be ruled out. An ultrasound-guided fine-needle aspiration (FNA) was performed to obtain material for cytological evaluation. During the procedure, it was possible to aspirate fluid from the lesion using a 21 gauge needle (diameter 0.8 mm) (Figure 2), yielding 10 cl of white, turbid material. Cytological evaluation revealed that the lesion was filled with predominantly keratinaceous debris, some squamous cells, a few lymphocytes and no atypical cells. This established the cytological diagnosis of an epidermal inclusion cyst in the thyroid.

## Follow-up

**Figure 3.**
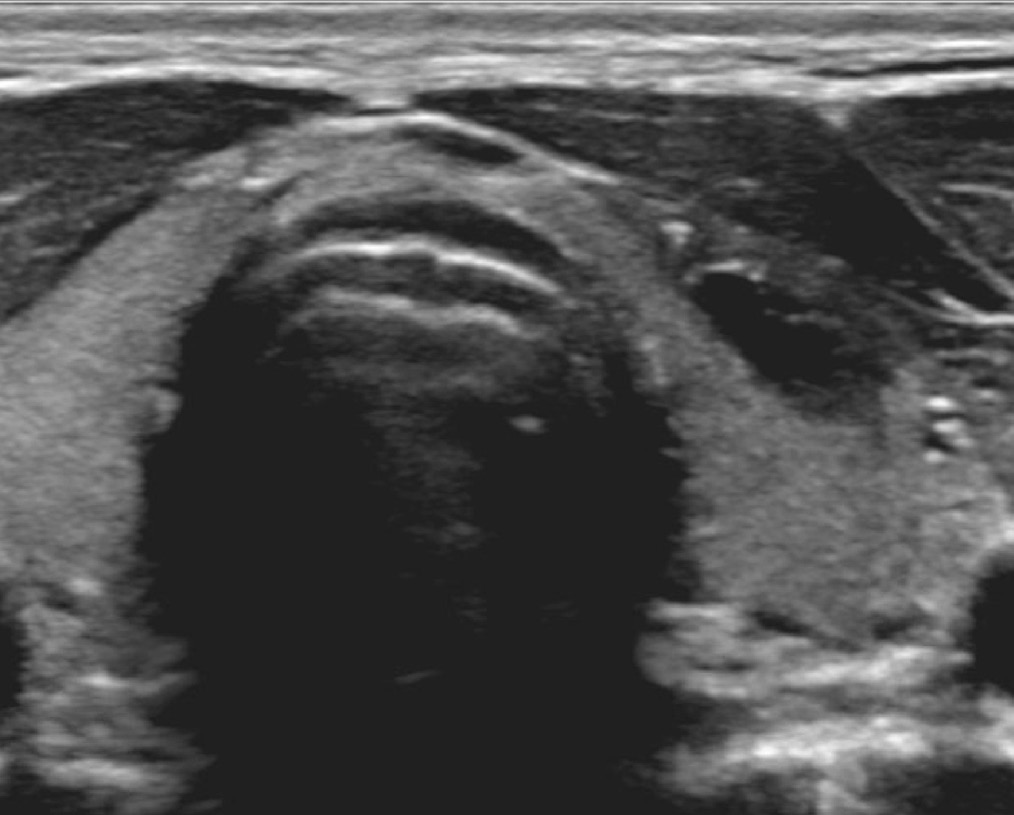
The cyst is filled with a small amount of hypoechoic fluid 3 months after aspiration.

The patient returned to our clinic 3 months later. The thyroidal mass had not returned and the patient did not report any complaints. Thyroid ultrasound (Figure 3) showed a residual thyroidal lesion of 1.1 cm wide, 0.5 cm deep and 2.5 cm tall, with calcifications in its wall. Dystrophic calcifications in cyst walls of benign epidermoid cysts of the head and neck have been described before.^1^ The contents of the cyst were markedly hypoechoic at follow-up. No abnormal lymph nodes were seen. The option to perform a hemithyroidectomy to remove the epidermal inclusion cyst and consequently perform histological analysis of the tissue was discussed with the patient and his parents. Due to the (probably) very low risk of malignancy in epidermal inclusion cysts and the potential postoperative morbidity (including recurrent laryngeal nerve injury, bleeding, infection and keloid development), the shared decision was made not to perform a hemithyroidectomy immediately.^2^ Follow-up by ultrasound evaluation once to twice a year was proposed.

## Discussion

Epidermal cysts of the thyroid are very rare. Less than 20 cases have been reported, of which only two involved a paediatric patient.^3–9^ The thyroid nodule-like appearance at sonographic evaluation may mimic the radiological characteristics of a solid thyroidal neoplasm. That is why cytological evaluation is essential in the diagnostic work up. Epidermal cysts mainly contain keratin and benign squamous cells. A resection and subsequently histological evaluation can be considered to ascertain a histological diagnosis. Epidermal cysts may be differentiated from dermoid cysts. Epidermal cysts arise from ectodermal germ cell tissue, while dermoid cysts additionally contain mesodermal tissue. A dermoid cyst may therefore be filled with hair or sweat glands (skin adnexa), whereas these are lacking in epidermal cysts.^10^

Sonographic findings that are generally associated with a higher chance of thyroid carcinoma are a taller-than-wide shape, lack of smooth margin, hypoechogenicity, calcifications, abnormal lymph nodes and a solitary nodule.^11,12^ Although certain ultrasound characteristics are more associated with benign or malignant disease, ultrasound only cannot single out a thyroid adenoma or carcinoma. Our patient had a solitary lesion with a taller-than-wide shape, hypoechogenicity, calcifications and an abnormal lymph node. The very smooth margin of the lesion of our patient pointed towards a benign cause; however, it did not fully exclude the possibility of a thyroid carcinoma. Still, these sonographic characteristics were reported in diagnostic studies to help distinguish between thyroid adenoma and carcinoma, and reported positive/negative predictive values do not apply to epidermal cysts. For example, calcifications are associated with malignant disease, but also frequently seen in walls of epidermal cysts (dystrophic calcifications) and inside epidermal cysts.^1,10^

In prior case reports, patients were surgically treated, either by simple excision of the cyst, or hemithyroidectomy. In the herein reported case, a hemithyroidectomy was not performed yet after shared decision-making with the patient and his parents. Ultrasound follow-up of the lesions will be scheduled on a regular basis. In case of growth of the cyst, or mechanical or cosmetic complaints (signs that are also associated with malignant transformation), the decision to perform a hemithyroidectomy will be reconsidered.^2^

## Learning points

1. An epidermal inclusion cyst of the thyroid is a rare differential diagnosis of a thyroid nodule-like structure seen at thyroidal ultrasound.
2. Sonographic findings associated with an epidermal cyst of the thyroid may be similar to those associated with thyroid carcinoma. Cytological evaluation is essential in the diagnostic work up to rule out a neoplasm.
